# Arterial stiffness and biological parameters: A decision tree machine learning application in hypertensive participants

**DOI:** 10.1371/journal.pone.0288298

**Published:** 2023-07-07

**Authors:** Alexandre Vallée

**Affiliations:** Department of Epidemiology and Public Health, Foch hospital, Suresnes, France; Osaka University of Pharmaceutical Sciences, JAPAN

## Abstract

Arterial stiffness, measured by arterial stiffness index (ASI), could be considered a main denominator in target organ damage among hypertensive subjects. Currently, no reported ASI normal references have been reported. The index of arterial stiffness is evaluated by calculation of a stiffness index. Predicted ASI can be estimated regardless to age, sex, mean blood pressure, and heart rate, to compose an individual stiffness index [(measured ASI–predicted ASI)/predicted ASI]. A stiffness index greater than zero defines arterial stiffness. Thus, the purpose of this study was 1) to determine determinants of stiffness index 2) to perform threshold values to discriminate stiffness index and then 3) to determine hierarchical associations of the determinants by performing a decision tree model among hypertensive participants without CV diseases. A study was conducted from 53,363 healthy participants in the UK Biobank survey to determine predicted ASI. Stiffness index was applied on 49,452 hypertensives without CV diseases to discriminate determinants of positive stiffness index (N = 22,453) from negative index (N = 26,999). The input variables for the models were clinical and biological parameters. The independent classifiers were ranked from the most sensitives: HDL cholesterol≤1.425 mmol/L, smoking pack years≥9.2pack-years, Phosphate≥1.172 mmol/L, to the most specifics: Cystatin c≤0.901 mg/L, Triglycerides≥1.487 mmol/L, Urate≥291.9 *μ*mol/L, ALT≥22.13 U/L, AST≤32.5 U/L, Albumin≤45.92 g/L, Testosterone≥5.181 nmol/L. A decision tree model was performed to determine rules to highlight the different hierarchization and interactions between these classifiers with a higher performance than multiple logistic regression (p<0.001). The stiffness index could be an integrator of CV risk factors and participate in future CV risk management evaluations for preventive strategies. Decision trees can provide accurate and useful classification for clinicians.

## Introduction

Arterial stiffness (AS), measured by arterial stiffness index (ASI), could be considered to be a main predictor in target damage of organs among primary hypertensive subjects [1]. AS is correlated with coronary atherosclerosis [2], cardiovascular (CV) diseases [3] and inflammatory processes [4]. Numerous investigations have observed that carotid-femoral (aortic) pulse wave velocity (PWV) can provide a standard measurement of AS. PWV levels are mainly associated with CV risk factors, including atherosclerosis [5], hypertension and diabetes [6], and CV events [7, 8]. ASI is a well-known and non-invasive method to estimate AS by performing infrared light (photoplethysmography) to record, in the finger, the volume waveform of the blood. The shape of the waveform is directly correlated to the time it takes for the pulse wave to travel in the arterial tree. These tools could be of interest to quickly estimate risk of CV diseases [9–16]. However, ASI, as the gold standard PWV, is not performed in routine clinical practice, face the difficulty in determining a nonpathological threshold value. Although some European consortiums, have reported normal references for PWV, as values under cut-off of 10m/s, but not for ASI. Thus, it remains difficult to interpret individually high values of ASI [7].

However, individually, only ASI measure shows no relevant information. Numerous factors were strongly associated with AS, including age, gender, blood pressure (BP), and heart rate (HR) [17]. An index of AS (called in this study: stiffness index) could be calculated and could be a higher predictor of personalized CV care. Firstly, a predicted ASI, based on these above factors, was performed to determine the personalized relevance of AS. Secondly, the stiffness index was calculated as ([measured ASI–predicted ASI]/predicted ASI) to classify individuals with increased AS or not, regardless of age, gender, mean BP, and HR. Although AS is a predictive factors of CV disease, the determinants of increased stiffness index have not been investigated, especially through a machine learning decision tree model and in a population of hypertensive participants without CV diseases. Hypertensive subjects are mainly at risk of future CV diseases [18].

Investigation of the potential determinants of increased AS in a large hypertensive population could provide a better understanding of consistent data to prevent CV risks. Machine learning, such as the decision tree, is a retrospective computational method to highlight information from a large dataset. Decision tree models could be one of the major algorithms in data mining tools for the management prevention of CV disease [19–21]. A decision tree model performs a tree-based hierarchization to generate a predictive tool based on predictive factors. It allows the added information of novel interactions between independent variables. The advantage of using a decision tree model is the ability to transform complex algorithms into a simple and organized flowchart, which can be used to present the hierarchy of determinants and to generate clinical and practice risk stratification tools to better manage diseases. The decision tree model allows clinicians to prioritize the different risk factors and to investigate their different interactions. A simple practical model can help health professionals to make more valid risk-based clinical decisions. Moreover, this machine learning tool can be used to decrease unnecessary factors selection with a view to better understand risk factors associated with arterial stiffness. Standard linear or logistic regression models fail in clinical topics where the associations between features and outcomes are nonlinear or where factors directly interact with each other. Decision tree models split the dataset in several times according to determined cutoff values. Thus, these types of models are simple and easy to understand, interpret and visualize. To date, few studies have focused on the determination of determinants of AS by this methodology.

Thus, the purpose of this study was 1) to determine determinants of stiffness index 2) to perform threshold values to discriminate stiffness index and then 3) to determine hierarchical associations of the determinants by performing a decision tree model among hypertensive participants without CV diseases.

## Methods

### UK Biobank population

The UK Biobank is a prospective cohort for the investigation, prevention, diagnosis and treatment of chronic diseases, such as CV diseases in adults. Between, 2006 and 2010, 502,478 Britons across 22 UK cities from the UK National Health Service Register were included. The cohort was phenotyped and genotyped, by participants who responded to a questionnaire; a computer-assisted interview; physical and functional measures; and blood, urine, and saliva samples [22]. Data included socio-economic factors, behaviour and lifestyle, a mental health battery, clinical diagnoses and therapies, genetics, imaging and physiological biomarkers from blood and urine samples. The cohort protocol can be found in the literature [23]. All participants provided electronic informed consent and UK Biobank received ethical approval from the North-West Multi-centre Research Ethics Committee (MREC) covering the whole of UK. The study was conducted in accordance with the guidelines of the Declaration of Helsinki, and approved by the North West–Haydock Research Ethics Committee (protocol code: 21/NW/0157, date of approval: 21 June 2021). For details https://www.ukbiobank.ac.uk/learn-more-about-uk-biobank/about-us/ethics.

### Blood pressure measurement

Systolic and diastolic blood pressures (SBD, DBP) were measured twice at the assessment centre by the use of an automated BP device (Omron 705 IT electronic blood pressure monitor; OMRON Healthcare Europe B.V. Kruisweg 577 2132 NA Hoofddorp), or manually by the use of a sphygmomanometer with an inflatable cuff in association with a stethoscope if the blood pressure device failed to measure the BP or if the largest inflatable cuff of the device did not fit around the individual’s arm [24].

The participant was sitting in a chair for all the measures. They were carried out by nurses trained in performing BP measures on the left upper arm [25]. Multiple available measures for each participant were averaged. The Omron 705 IT BP monitor has satisfied the Association for the Advancement of Medical Instrumentation SP10 standard and was validated by the British Hypertension Society protocol, with an overall “A” grade for both SBP and DBP [26]. Nevertheless, automated devices measure higher BP in comparison to manual sphygmomanometers, thus, adjusted both SBP and DBP were measured using the automated device using algorithms by Stang et al. [27]:

For SBP, the following algorithm was performed:

$$SBP=3.3171+0.92019\times SBP\;\left(mmHg\right)+6.02468\times sex\;\left(male=1;female=0\right)$$

For DBP, the following algorithm was performed:

$$DBP=14.5647+0.80929\times DBP\;\left(mmHg\right)+2.01089\times sex\;\left(male=1;female=0\right)$$

These adjusted BP values were used for all calculations, including mean BP calculation [28].

Mean BP was calculated as:

$$mean\;BP=\left(\left(SBP+2\times DBP\right)\right)/3$$

### Outcomes

Pulse wave arterial stiffness index (ASI) was estimated through a non-invasive method during a volunteer’s visit to a UK Biobank Assessment Centre. Peripheral blood volume was taken by clipping a photoplethysmograph transducer (PulseTrace PCA 2^™^, CareFusion, USA) to the rested volunteer’s finger (preferably the index finger of the non-dependent hand although it can be placed on any finger). Volunteers were asked to breathe in and out slowly five times in a relaxed fashion and readings were taken over 10–15 seconds. The carotid-to-femoral pulse transit time was estimated from the dicrotic waveform as the time difference between a forward compound when the pressure is transmitted from the left ventricle to the finger and a reflected or backward compound as the wave is transmitted from the heart to lower body via the aorta [29]. ASI was estimated in metres per second (m/s) as: H/PTT. H is the individual’s height, and PTT is the pulse transit time or the peak-to-peak time between the systolic and diastolic wave peaks in the dicrotic waveform [29]. This methodology has been validated by three independent studies comparing it with carotid femoral PWV. These studies concluded that both measure methods were highly correlated. ASI was a simple, operator independent, non-expensive and rapid method [10, 29–31]. Extreme outlier ASI values were excluded from the analyses (defined as mean +/- 5*standard deviation) [28, 32].

### Laboratory and clinical parameters

Hypertension was defined as systolic blood pressure (SBP) of at least 140 mm Hg and/or diastolic BP (DBP) at least 90 mm Hg, according to guidelines by the European Society of Cardiology, and/ or antihypertensive drug used [33], or hypertension diagnosed by a doctor. Diabetes status was based on either receiving anti-diabetic medication or diabetes diagnosed by a doctor or a fasting glucose concentration ≥7mmol/L. Dyslipidemia was defined as having a fasting plasma total-cholesterol≥6.61 mmol/L or LDL cholesterol ≥4.1 mmol/L or triglycerides level>1.7 mmol/ or taking statins medication. Estimated glomerular filtration rate (eGFR) was calculated based on the Chronic Kidney Disease Epidemiology Collaboration equation (eGFR-CKD-EPI), as follows:

eGFR=141×minimumof1orstandardizedScr/κ^α×maximumof1orstandardizedScr/κ^−1.209×〖0.993〗^age×1.018iffemale

where κ is 0.7 in females and 0.9 in males, and α is −0.329 in females and −0.411 in males. e-GFR <60 mL/min/1.73 m^2^ defined chronic kidney disease (CKD)). Current tobacco smokers were defined as participants who responded “yes, on most or all days” at the question “do you smoke tobacco now”. Smoking pack-years are calculated by physicians during examination as the reported average number of smoking packs per day divided by the total number of years of smoking during their lifetime, for never-smokers the value was zero. CV diseases were defined by heart attack, angina and stroke, as diagnosis by a doctor and reported in questionnaires. Body mass index was calculated as weight (in kg) divided by height^2^ (metres).

### Study population

**Fig 1:**
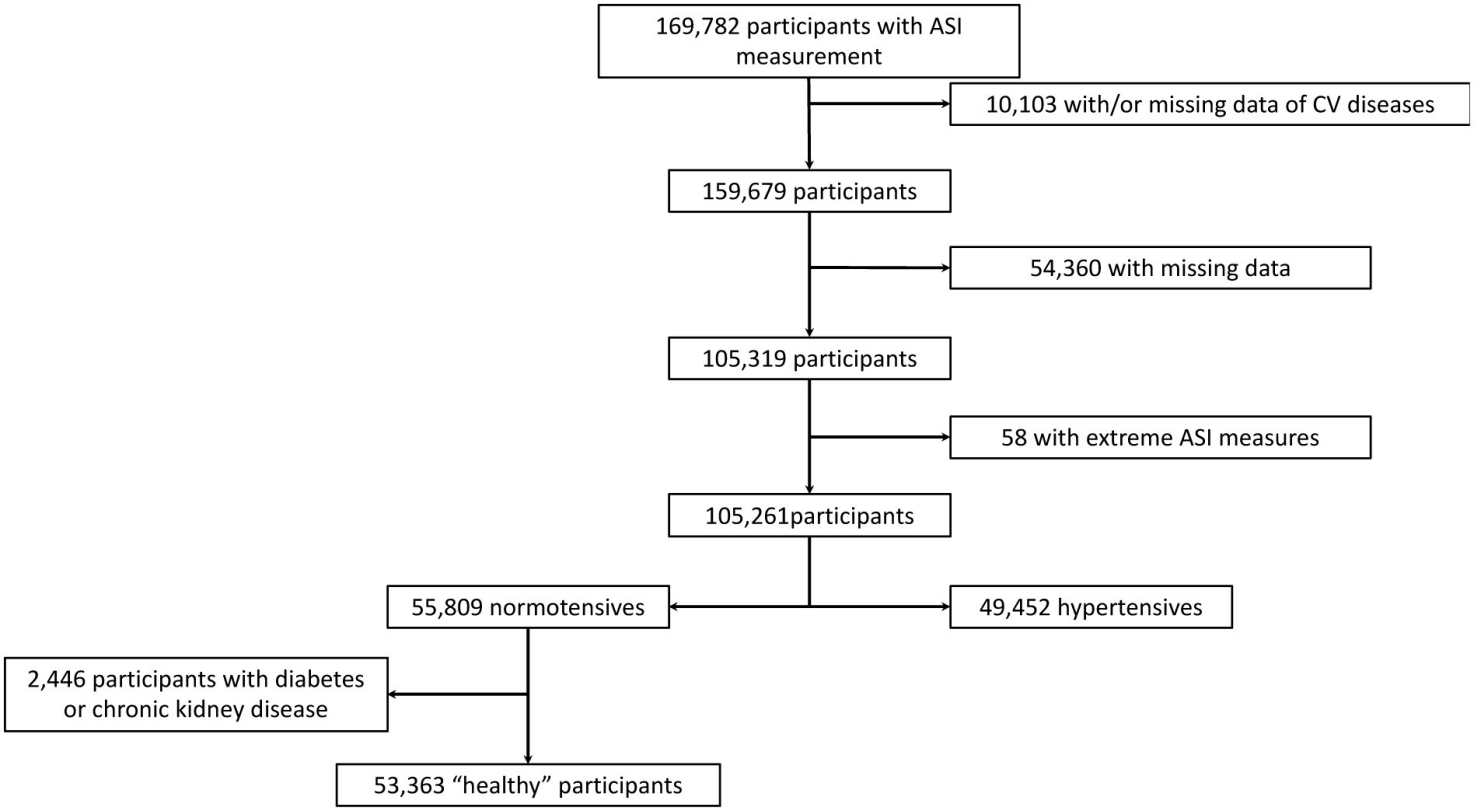
Flowchart.

‘Healthy’ participants used for the determination of stiffness index were defined as individuals without hypertension, without diabetes mellitus, without previous cardiovascular events and without chronic kidney disease (N = 53,363) to performed predicted ASI. All hypertensive participants without cardiovascular diseases were included in this work (N = 49,452) (Fig 1) to investigate all the possible factors of AS.

### Determination of the stiffness index based on healthy participants

#### Equation of predicted ASI

Among the 53,363 “healthy” participants, parameters which modulate ASI can be evaluated independently of gender, age, mean BP and HR. A stiffness index was performed by a multivariate linear regression analysis to determine predicted ASI values based on age, gender (male = 1, female = 0), mean BP and HR. In the multivariate linear regression, all these parameters were significant (p<0.001). Then, an equation was derived from the multiple linear regression and was applied to the individuals to perform a predicted ASI value according to their age, gender, mean BP and HR.

#### Equation of the stiffness index

The stiffness index was defined as:

$$\text{Stiffness}\;\text{index}=\left(measured\;ASI-predicted\;ASI\right)/\left(predicted\;ASI\right)$$

Increased arterial stiffness was defined when stiffness index was greater than 0.

#### Decision tree model

The target or outcome variable consisted in two classes: one class for the positive stiffness index (value>0) and the second for negative stiffness index (value <0). Data mining detects unknown patterns or prediction rules. One of the different methods of data mining is the decision tree. The decision tree model is a non-parametric methodology which performed a tree-based classification modeling [20, 21]. The main purpose of this methodology is to provide a predictive tool for the target interested variable regardless of predictors. Decision tree models are composed by three types of nodes: root node, internal node, and end node [34]. This methodology performs splitting criteria to break a node to form a tree. The internal variables of the model represent a tree structure in which a decision is performed on each branch according to the data features. Splitting criteria provide a rate for each predictive variable. Variables that have the best rate of splitting criteria are selected as staying in the algorithm. In the decision tree, the first variable or root node is the main important determinant and then, the other variables could be classified in order of importance [35]. The root node is the variable that can divide the whole population with the highest information gain.

The Classification And Regression Tree (CART) is a decision tree algorithm [36]. CART is made by splitting subsets of data using all predictor variables. By this procedure, all root nodes are created repeatedly. The CART algorithm creates a binary division of the tree and pruning a tree on the cost complexity [37]. The CART algorithm uses the Gini impurity index to select the best variable.

Impurity was measured by the Gini index as:

$$Gini\;\left(D\right)=1-\overset{m}{\underset{i=1}{\sum }}P_{i}^{2}$$

Then, this data mining method decision tree randomly divides the dataset into one hundred models which have been trained for the repeated cross-validation procedure to obtain the accuracy of the model. Data mining algorithms, and particularly the decision tree, do not work with missing data. Therefore, after cleaning and preparing the dataset, 49,452 hypertensive participants without CV diseases were included in the final data analysis.

### Statistical analysis

Characteristics of the study population were described as the means with standard deviation (SD) for continuous variables. Comparisons between groups were performed using Student’s test for continuous variables. Pearson’s Chi2 test was performed for categorical variables. A multivariate linear regression analysis was performed with age, sex, mean BP and HR to determine the equation of the predicted ASI. Then, a forward-backward logistic multivariate regression analysis model was performed based on univariate significant covariates (p<0.05) for the difference between positive and negative stiffness index.

For each independent classifier of the logistic multivariate analysis, the ability of the logistic regression models to allow discrimination was quantified by the area under the ROC curve (AUC).

The maximum Youden index, performed as:

$$J=max_{c}\left[S_{e}\left(c\right)+S_{p}\left(c\right)-1\right]$$

was chosen to determine the optimal decision thresholds (*c*) for discrimination. The thresholds of the independent variables of this logistic multivariate analysis were considered as input variables in the decision tree model. A confusion matrix was utilized to determine the performance of the decision tree process for the discrimination procedure. The accuracy and the receiver operating characteristics (ROC) curve was measured [38]. The ROC graph is a method for visualizing and selecting classifiers based on their performance [39]. The area under the curve (AUC) of the classifier can be described as the probability of the classifier to rank a randomly selected positive results in higher predictive accuracy [40]. Comparison of the model performances between logistic regression model and decision tree algorithm was performed through the DeLong test [41]. Statistics were performed using SAS software (version 9.4; SAS Institute, Carry, NC). A P value <0.05 was considered statistically significant.

## Results

Among the 53,363 “healthy” participants, stiffness index was performed by a linear regression analysis to determine predicted ASI values based on age, gender (male = 1, female = 0), mean BP and HR (Table 1).

**Table 1:** Multivariate linear regression among healthy participants to determine predicted ASI based on age, gender, mean BP and HR.

Parameters	Beta coefficient (SE)	P value
Intercept	-1.167 (0.172)	<0.001
Age (years)	0.0837 (0.001)	<0.001
Gender (male = 1, female = 0)	0.9337 (0.247)	<0.001
HR (bpm)	0.0203 (0.001)	<0.001
Mean BP (mmHg)	0.0402 (0.002)	<0.001

Thus, predicted ASI was performed by the following algorithm:

$$\begin{array}{l} Predicted\;ASI=-1.167+0.0837\times age\;\left(year\right)+0.9337\times sex\;\left(male=1;female=0\right)\\ +0.0402\times mean\;BP\left(mmHg\right)+0.0203\times HR\;\left(bpm\right) \end{array}$$

Then, stiffness index was calculated in the hypertensive population based on the determination of predicted ASI. Hypertensive participants showed a mean stiffness index at 0.0163 (0.3116) compared to value of stiffness index equal to zero for healthy participants (p<0.001) (Table 2). The characteristics of the 53,363 “healthy” participants and of the 49,452 hypertensive participants were shown in Table 2. “Healthy participants” were significantly different compared to the hypertensive participants for all covariates, except for lipoprotein (a) (p = 0.113). Values of ASI were 8.8m/s (SD 2.8) for healthy participants compared to 9.9m/s (SD 3.2) for the hypertensive participants (p<0.001) with 12.6m/s (SD 2.3) for positive stiffness index and 7.6m/s (SD 1.7) for negative stiffness index (p<0.001). Values of stiffness index were 0.26 (SD 0.22) for positive stiffness index and -0.24 (SD 0.16) for negative stiffness index (p<0.001). The hypertensive participants were divided into the two groups (Positive stiffness index, N = 22,453 (45.4%) and Negative stiffness index, N = 26.999 (54.6%)) are shown in Table 3. Among hypertensive participants, positive stiffness index participants did not differ for antidiabetic therapy (p = 0.846), antihypertensive therapy (p = 0.582), statins (p = 0.648), and diabetes (p = 0.401). For biological and blood parameters, the two groups differed except for total cholesterol (p = 0.073), serum creatine (p = 0.080), LDL cholesterol (p = 0.070) and Lipoprotein (a) (p = 0.124). Collinearities between all biological parameters were shown in S1 Table.

**Table 2:** Clinical, biological and hemodynamic characteristics of the study population.

	Healthy participants		Hypertensive participants		
	N = 53,363		N = 49,452		P value
Gender					<0.001
male	21587	40.45%	29866	60.39%	
female	31776	59.55%	19586	39.61%	
Age (years)	53.71	8.1778	58.91	7.433	<0.001
Body Mass index (BMI), Kg/m2	26.154	4.1404	28.728	4.9115	<0.001
Systolic Blood Pressure (SBP), mmHg	121.81	10.904	145.65	15.188	<0.001
Diastolic Blood Pressure (DBP), mmHg	77.93	6.0683	87.13	7.731	<0.001
Mean Blood Pressure (MBP), mmHg	92.558	6.9928	106.639	8.889	<0.001
Heart Rate (HR), bpm	66.931	9.8395	70.088	11.917	<0.001
Arterial Stiffness index (ASI), m/s*	8.7923	2.7573	9.8722	3.1864	<0.001
Stiffness index**	-	-	0.0163	0.3116	<0.001
Current tobacco (yes)	4251	7.97%	3220	6.51%	<0.001
Smoking pack years (pack-years)	5.1724	11.634	7.5196	15.563	<0.001
Cancer	4135	7.75%	4364	8.82%	<0.001
Autoimmune diseases	2866	5.37%	3428	6.93%	<0.001
Depression	2854	5.35%	2298	4.65%	<0.001
Dyslipidemia	24864	46.59%	33915	68.58%	<0.001
Diabetes	-	0.00%	4890	9.89%	<0.001
Antidiabetics	-	0.00%	2762	5.59%	<0.001
Statins	2897	5.43%	12466	25.21%	<0.001
Anti-hypertensive therapy	-	-	20190	40.83%	<0.001
Chronic Kidney disease (CKD)	-	-	1978	4.00%	<0.001
Glomerular filtration rate (GFR), mL/min/1.73 m^2^	120.3	33.256	107.6	33.400	<0.001
Alanine Aminotransferase (ALT), U/L	21.109	12.002	25.431	14.740	<0.001
Albumin, g/L	45.4	2.5823	45.5	2.659	<0.001
Alkaline Phosphatase, U/L	80.04	23.639	85.64	24.752	<0.001
Apolipoprotein A1, g/L	1.5567	0.2654	1.5247	0.2641	<0.001
Apolipoprotein B, g/L	1.0198	0.2063	1.0274	0.2197	<0.001
Aspartate aminotransferase (AST), U/L	25.024	8.7137	27.362	10.1514	<0.001
Calcium, mmol/L	2.3805	0.0912	2.3933	0.0960	<0.001
Creatine, micromole/L	70.421	12.701	75.608	19.829	<0.001
C reactive protein (CRP), mg/L	2.0883	3.7059	2.8707	4.4269	<0.001
Cystatin c, mg/L	0.8638	0.1242	0.9397	0.1864	<0.001
Gamma glutamyl transferase, U/L	30.354	30.944	41.992	43.386	<0.001
Glucose, mmol/L	4.9455	0.5004	5.3319	1.2837	<0.001
HbA1c, mmol/L	34.484	3.8244	37.128	7.6099	<0.001
HDL cholesterol, mmol/L	1.4959	0.3652	1.3971	0.3573	<0.001
LDL cholesterol, mmol/L	3.5505	0.7239	3.4987	0.7935	<0.001
Total cholesterol, mmol/L	5.6879	0.9294	5.5862	1.0392	<0.001
Triglycerides, mmol/L	1.533	0.8623	1.8527	1.0037	<0.001
Lipoprotein (a), nmol/L	44.172	48.565	44.712	49.312	0.113
Insulin-like Growth Factor (IGF), nmol/L	22.189	5.5118	21.153	5.6732	<0.001
Phosphate, mmol/L	1.1973	0.1537	1.1612	0.1607	<0.001
Total bilirubin, micromol/L	9.2611	4.522	9.5282	4.455	<0.001
Testosterone, nmol/L	5.8239	6.2163	7.5242	5.9183	<0.001
Urate, mmol/L	289.72	72.559	33.20	79.401	<0.001
Vitamin D, nmol/L	50.576	21.329	49.348	21.011	<0.001

* measured by a non-invasive method during volunteer’s visit

**calculated based on the multivariate linear regression

**Table 3:** Clinical, biological, and hemodynamic characteristics of the hypertensive population according to the stiffness index (negative or positive).

	Hypertensive participants				
	Negative stiffness index		Positive stiffness index		
	N = 26,999		N = 22,453		P value
Gender					<0.001
male	15871	58.78%	13995	62.33%	
female	11128	41.22%	8458	37.67%	
Age (years)	59.098	7.5358	58.698	7.3026	<0.001
Body Mass index (BMI), Kg/m2	28.513	5.0723	28.989	4.6981	<0.001
Systolic Blood Pressure (SBP), mmHg	146.49	15.485	144.65	14.762	<0.001
Diastolic Blood Pressure (DBP), mmHg	86.827	7.7218	87.5	7.7272	<0.001
Mean Blood Pressure (MBP), mmHg	106.71	8.9347	106.55	8.8336	0.039
Heart Rate (HR), bpm	70.354	12.659	69.767	10.952	<0.001
Arterial Stiffness index (ASI), m/s*	7.6314	1.7449	12.567	2.3242	<0.001
stiffness index**	-0.2410	0.1573	0.2552	0.2231	<0.001
Current tobacco (yes)	1418	5.25%	1802	8.03%	<0.001
Smoking pack years (pack-years)	6.6638	14.66	8.5486	16.525	<0.001
Cancer	2382	8.82%	1982	8.83%	0.985
Autoimmune disorders	1829	6.77%	1599	7.12%	0.130
Depression	1195	4.43%	1103	4.91%	0.011
Dyslipidemia	18083	66.98%	15832	70.51%	<0.001
Diabetes	2642	9.79%	2248	10.01%	0.401
Antidiabetics	1503	5.57%	1259	5.61%	0.846
Statins	6784	25.13%	5682	25.31%	0.648
Anti-hypertensive therapy	11053	40.94%	9137	40.69%	0.582
Chronic Kidney disease (CKD)	1125	4.17%	853	3.80%	0.038
Glomerular filtration rate (GFR), mL/min/1.73 m^2^	107.88	33.679	107.26	33.059	0.039
Alanine Aminotransferase (ALT), U/L	24.683	14.157	26.332	15.364	<0.001
Albumin, g/L	45.597	2.6877	45.459	2.624	<0.001
Alkaline Phosphatase, U/L	85.375	25.597	85.964	23.698	0.009
Apolipoprotein A1, g/L	1.5408	0.2694	1.5029	0.2562	<0.001
Apolipoprotein B, g/L	1.0229	0.2194	1.0333	0.2199	<0.001
Aspartate aminotransferase (AST), U/L	27.24	10.126	27.509	10.18	0.004
Calcium, mmol/L	2.3943	0.0961	2.3922	0.0959	0.014
Creatine, micromole/L	75.466	19.814	75.78	19.846	0.080
C reactive protein (CRP), mg/L	2.7613	4.4469	2.9062	4.4017	<0.001
Cystatin c, mg/L	0.937	0.1926	0.9431	0.1787	<0.001
Gamma glutamyl transferase, U/L	40.814	42.79	43.41	44.053	<0.001
Glucose, mmol/L	5.3497	1.272	5.3105	1.2974	<0.001
HbA1c, mmol/L	37.016	7.6497	37.252	7.5601	<0.001
HDL cholesterol, mmol/L	1.4255	0.3663	1.3628	0.3431	<0.001
LDL cholesterol, mmol/L	3.4928	0.7963	3.5057	0.7902	0.070
Total cholesterol, mmol/L	5.5939	1.045	5.5771	1.032	0.073
Triglycerides, mmol/L	1.7829	0.9688	1.9367	1.0382	<0.001
Lipoprotein (a), nmol/L	44.364	49.053	45.131	49.622	0.124
Insulin-like Growth Factor (IGF), nmol/L	21.205	5.7094	21.092	5.629	0.029
Phosphate, mmol/L	1.1554	0.1592	1.1684	0.1622	<0.001
Total bilirubin, micromol/L	9.5901	4.5324	9.4561	4.3604	<0.001
Testosterone, nmol/L	7.3669	5.9515	7.7134	5.8726	<0.001
Urate, mmol/L	329.92	80.485	337.14	77.897	<0.001
Vitamin D, nmol/L	49.696	21.202	48.93	20.772	<0.001

* measured by a non-invasive method during volunteer’s visit

**calculated based on the multivariate linear regression

### Determinants of stiffness index

The ten remaining independent classifiers of the forward-backward multivariate model were Alanine aminotransferase (ALT) (p<0.001), Albumin (p<0.001), Testosterone (p = 0.001), Phosphate (p<0.001), Aspartate aminotransferase (AST) (p<0.001), Cystatin c (p<0.001), smoking pack years (p<0.001), HDL cholesterol (p<0.001), Triglycerides (p<0.001) and Urate (p = 0.004) (Table 4).

**Table 4:** Forward-backward multivariate logistic regression model for positive stiffness index discrimination among hypertensive participants.

Parameters	Estimate (SE)	P value	Threshold	AUC	P value
Alanine Aminotransferase (ALT), U/L	0.01 (0.001)	<0.001	22.13	0.540	<0.001
Albumin, g/L	-0.03 (0.004)	<0.001	45.92	0.516	<0.001
Testosterone, nmol/L	0.006 (0.001)	0.001	5.181	0.518	0.001
Phosphate, mmol/L	0.72 (0.06)	<0.001	1.172	0.523	<0.001
Aspartate aminotransferase (AST), U/L	-0.01 (0.001)	<0.001	32.5	0.508	<0.001
Cystatin c, mg/L	-0.23 (0.06)	<0.001	0.901	0.521	<0.001
Smoking pack years	0.01 (0.001)	<0.001	9.2	0.530	<0.001
HDL cholesterol, mmol/L	-0.31 (0.03)	<0.001	1.425	0.551	<0.001
Triglycerides, mmol/L	0.07 (0.01)	<0.001	1.487	0.549	<0.001
Urate, mmol/L	0.001 (0.0001)	0.004	291.9	0.530	<0.001

### Thresholds of the determinants of stiffness index

By calculating the Youden index, this study could determine the thresholds for each independent classifier for the maximum discrimination rate. Threshold for ALT: ≥22.13 U/L, for Albumin: ≤45.92 g/L, for Testosterone: ≥5.181 nmol/L, for Phosphate: ≥1.172 mmol/L, for AST: ≤32.5 U/L, for Cystatin c: ≤0.901 mg/L, for smoking pack years: ≥9.2 packs-years, for HDL cholesterol: ≤1.425 mmol/L, for Triglycerides: ≥1.487 mmol/L, and for Urate: ≥291.9 *μ*mol/L (Table 4).

### Decision tree model for hierarchization of the determinants of stiffness index

The if-then rules created by the model for the discrimination of positive stiffness index participants are shown in Table 5.

**Table 5:** Rules extracted through the decision tree model for discriminate positive from negative stiffness index participants among hypertensive participants. The percentages expressed the proportion of positive stiffness index in each subgroup.

Rules	First step of the Rule	First number and proportion of positive stiffness index of the Rule	All steps of the Rule	Final number and proportion of positive stiffness index of the Rule
Rule 1	If, HDL cholesterol≤1.425 mmol/L	N = 29,075, 48.6%	If, HDL cholesterol≤1.425 mmol/L, smoking pack years≥9.2pack-years, Phosphate≥1.172 mmol/L, Cystatin c≤0.901 mg/L, Triglycerides<1.487 mmol/L, ALT≥22.13 U/L, AST>32.5 U/L, and Testosterone≥5.181 nmol/L	N = 40, 70.0%
Rule 2	If, HDL cholesterol>1.425 mmol/L	N = 20,377, 40.8%	If, HDL cholesterol>1.425 mmol/L, smoking pack years≥9.2pack-years, Triglycerides≥1.487 mmol/L, Phosphate<1.172 mmol/L, Urate<291.9 *μ*mol/L, Testosterone≥5.181 nmol/L, AST≤32.5 U/L, Cystatin c>0.901 mg/L	N = 22, 81.8%
Rule 3	If, HDL cholesterol≤1.425 mmol/L	N = 29,075, 48.6%	If, HDL cholesterol≤1.425 mmol/L, smoking pack years≥9.2pack-years, Phosphate<1.172 mmol/L, ALT<22.13 U/L, Urate<291.9 *μ*mol/L, Albumin>45.92 g/L, Cystatin c>0.901 mg/L, Triglycerides<1.487 mmol/L	N = 17, 82.4%
Rule 4	If, HDL cholesterol≤1.425 mmol/L	N = 29,075, 48.6%	If, HDL cholesterol≤1.425 mmol/L, smoking pack years≥9.2pack-years, Phosphate<1.172 mmol/L, ALT<22.13 U/L, Urate<291.9 *μ*mol/L, Albumin≤45.92 g/L, Cystatin c≤0.901 mg/L, Testosterone<5.181 nmol/L, Triglycerides≥1.487 mmol/L	N = 37, 80.1%
Rule 5	If, HDL cholesterol≤1.425 mmol/L	N = 29,075, 48.6%	If, HDL cholesterol≤1.425 mmol/L, smoking pack years≥9.2pack-years, Phosphate≥1.172 mmol/L, Cystatin c≤0.901 mg/L, Triglycerides≥1.487 mmol/L, Urate≥291.9 *μ*mol/L, ALT≥22.13 U/L, Testosterone≥5.181 nmol/L, AST≤32.5 U/L, Albumin>45.92 g/L	N = 129, 78.3%
Rule 6	If, HDL cholesterol>1.425 mmol/L	N = 20,377, 40.8%	If, HDL cholesterol>1.425 mmol/L, smoking pack years<9.2pack-years, ALT≥22.13 U/L, Triglycerides≥1.487 mmol/L, Albumin≤45.92 g/L, Urate<291.9 *μ*mol/L, Cystatin c>0.901 mg/L, Testosterone≥5.181 nmol/L	N = 24, 75.0%
Rule 7	If, HDL cholesterol>1.425 mmol/L	N = 20,377, 40.8%	If, HDL cholesterol>1.425 mmol/L, smoking pack years<9.2pack-years, Phosphate≥1.172 mmol/L, Urate≥291.9 *μ*mol/L, ALT≥22.13 U/L, Albumin≤45.92 g/L, AST<32.5 U/L, Testosterone≥5.181 nmol/L	N = 53, 77.3%
Rule 8	If, HDL cholesterol>1.425 mmol/L	N = 20,377, 40.8%	If, HDL cholesterol>1.425 mmol/L, smoking pack years≥9.2pack-years, Triglycerides<1.487 mmol/L, Testosterone<5.181 nmol/L, albumin 1, Urate≥291.9 *μ*mol/L, ALT≥22.13 U/L, Phosphate≥1.172 mmol/L, AST≤32.5 U/L, Cystatin c>0.901 mg/L	N = 20, 75.0%

**Fig 2:**
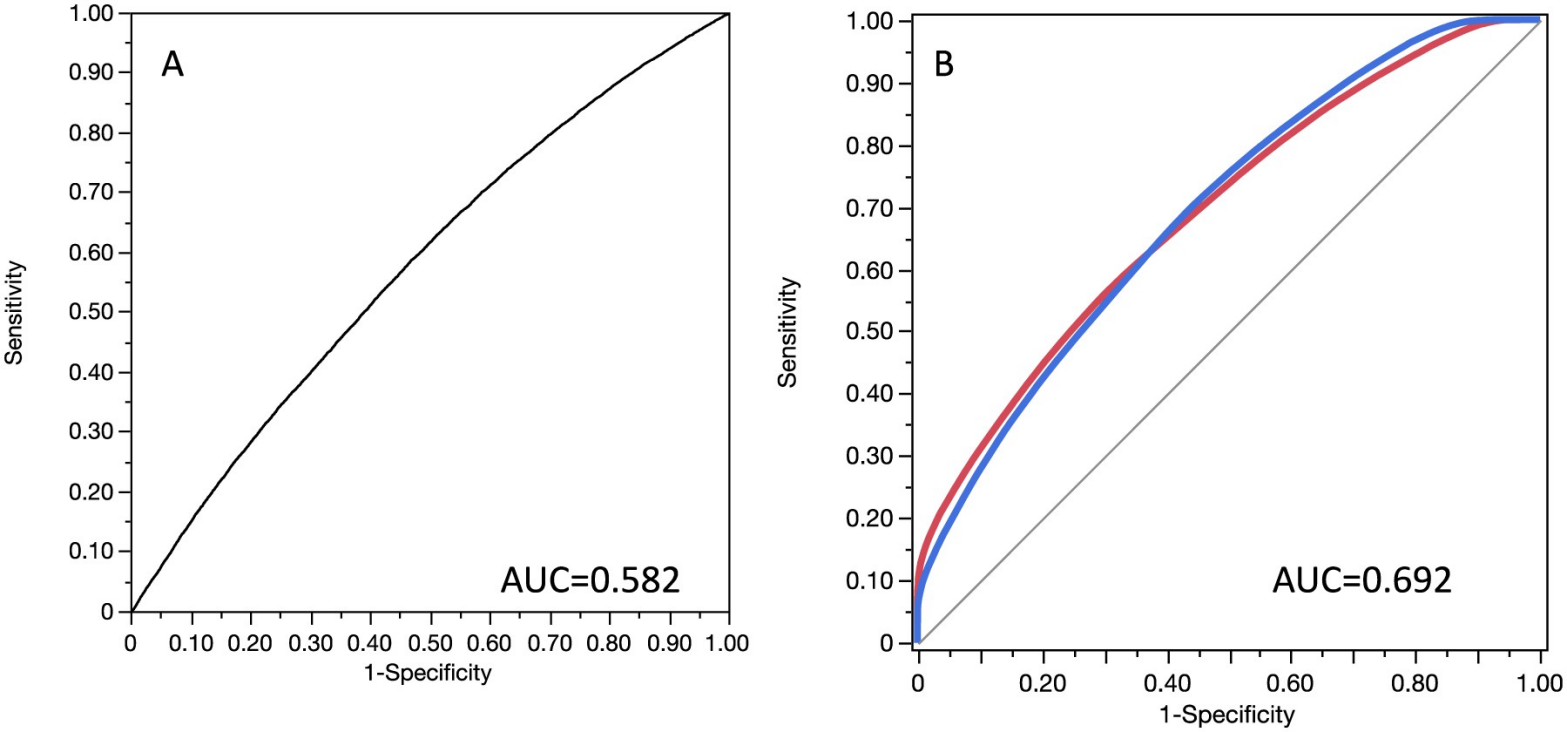
ROC curves with AUC (area under the ROC curve) for the multivariate logistic regression (A) and for the decision tree model (B).

AUC of ROC curve (AUC = 0.692) was obtained by applying one-hundred-fold cross validation (Fig 2). The performance, expressed by the AUC, of the Decision tree model was significantly higher than the performance of the logistic regression model (AUC = 0.692 vs AUC = 0.582, p<0.001).

The decision tree model 8 rules were selected with minimal positive stiffness index rate greater than 70% and a minimal population of 20 participants for the latest node.

The decision tree model presented that in a subgroup (rule 3) of smokers superior to 9.2 smoking pack-years with low levels of HDL cholesterol, Phosphate, ALT, Urate, and Triglycerides but high levels of Albumin and Cystatin C, the probability of positive stiffness index was 82.4%. In the subgroup (rule 2) of smokers superior to 9.2 pack-years with high levels of HDL cholesterol, Triglycerides, Testosterone and Cystatin c but low levels of Phosphate, Urate and AST, the probability of having a positive stiffness index was 81.8%. In the subgroup (rule 4) of smokers superior to 9.2 pack-years, with low levels of both HDL cholesterol, Phosphate, ALT, Urate, Albumin, Cystatin C and Testosterone, but with high levels of Triglycerides, the participants presented a probability of positive stiffness index at 80.1% (Table 5).

## Discussion

A stiffness index was performed based on a predicted ASI calculated by a linear regression model in an “healthy” with age, gender, mean BP and HR [20, 42, 43].

### Decision tree model application

A decision tree model was performed to investigate the interaction of the different thresholds of independent factors correlated with stiffness index (negative or positive values). A decision tree is a machine learning methodology that has several advantages, such as the ability to handle nonlinear relationships, creating rules, and being easy to interpret [44, 45]. None of these models have been performed to investigate the different determinants of stiffness index or the ASI values. One of the main added information in this study is the statistical comparison between the decision tree model and multivariate logistic regression. This study showed a higher performance of the data mining model compared to the standard statistical model.

The large sample of the UK Biobank allows us to add different biological parameters which are not measured in routine clinical practice for arterial stiffness, such phosphate and Cystatin C. Thus, by these results, the interest of decision trees is double: showing non-linear relationship and hierarchization of covariates and a higher accuracy compared to standard statistical models. Ten independent factors were highlighted in the logistic multivariate analysis. The ten independent classifiers were ranked from the most sensitives: HDL cholesterol≤1.425 mmol/L, smoking pack years≥9.2pack-years, Phosphate≥1.172 mmol/L, to the most specifics: Cystatin c≤0.901 mg/L, Triglycerides≥1.487 mmol/L, Urate≥291.9 *μ*mol/L, ALT≥22.13 U/L, AST≤32.5 U/L, Albumin≤45.92 g/L, Testosterone≥5.181 nmol/L.

### Classification tool for determinants of stiffness index

One key of the decision tree model was the classification tool of the different factors. The main sensitive factors (i.e. HDL cholesterol and smoking pack years) appeared at the beginning of the decision tree model, and at the end the main specific factors (exp. Urate, Cystatin c and Testosterone). For example, smokers superior to 9.2 smoking pack-years with low levels of HDL cholesterol, Phosphate, ALT, Urate, and Triglycerides but high levels of Albumin and Cystatin C, the probability of positive stiffness index was 82.4%. Thus, the decision tree model can present a probability based on a hierarchical classification of the different factors. To date, there are no investigation in the literature which used decision tree models for stiffness index or ASI values, and thus, it remains complicated to compare with other studies. Nevertheless, numerous investigations have observed that the eleven input variables remaining in the decision tree model have been mainly associated with arterial stiffness.

The two first-order factors (i.e. HDL cholesterol and Tobacco) are well-known factor of modulation of arterial stiffness [46].

### Tobacco and stiffness index

Several investigations have shown the potential relationship between tobacco use and AS [47]. In this study, we showed that tobacco smoking duration (expressed by a cutoff superior to 9.2 pack-years) is correlated with high ASI levels in a large sample. Current tobacco smoking is associated with AS [48], showing to the idea of that current smoking is correlated with both increased risk of atherosclerosis, reduction in arterial dilatation [48] and increase in muscular arteries [49].

### HDL-cholesterol and stiffness index

HDL-cholesterol possesses anti-CV role [50]. Several investigations have observed that the augmentation in HDL-cholesterol is associated with the diminution in of coronary heart disease risk [51]. Studies have observed that HDL-cholesterol was negatively associated with increase in AS [52]. HDL-cholesterol presents anti-atherosclerotic and non-atheromatous roles on the arterial wall which can decrease the stiffness of arteries [53]. Furthermore, HDL-cholesterol could have anti-inflammatory actions [21, 54].

### Triglycerides and stiffness index

Moreover, triglycerides was a major determinants for AS [55, 56]. Augmentation in triglycerides levels can enhance atherosclerosis by the scavenger receptor class B Type 1 (SR-BI) and by damaging capacity of HDL to secrete cholesteryl esters [57]. Augmentation rates of Triglycerides could participate in the involvement of inflammatory processes and oxidative stress to stimulate the production of adhesion molecule and the formation of foam cells, this to enhance the toxicity of smooth muscle [58] and to activate the release of endothelin-1, which is responsible for the development of atherosclerosis [59].

In parallel to HDL cholesterol, Triglycerides and tobacco smoking, this study presents other biological parameters as independent predictor of increased stiffness index, such as Urate, AL, AST, Albumin, and Phosphate. The interest of this work is to present these interesting factors and their interactions on each other in a classification tool, a decision tree model. In this study, these biological parameters are mainly associated with increased stiffness index than traditional factors, such as BMI, total cholesterol, diabetes, glycemia or kidney function [42]. This work could suggest adding these biological measures in a CV assessment faced with an increased AS suspicion and CV risks for the hypertensive population.

### Urate and stiffness index

Several findings have shown the association between Urate and AS in hypertensive subjects, while the mechanisms involved remains complex and not completely elucidated [60–62]. Urate can lead to oxidative stress, endothelial dysregulation, inflammation and fibrosis [62]. Urate-induced oxidative stress stimulates the production of endothelin-1 [63] and activated TGF-beta1 which leads to the production of proteoglycan, fibronectin and collagen synthesis responsible for increased AS [64].

### ALT and AST with stiffness index

Few studies have studied the association between arterial stiffness and serum ALT and AST [65–67]. AST and ALT are biomarkers reflecting disease severity in chronic liver diseases. A recent study has observed that the ratio AST/ALT is correlated with high risk of CV diseases for both genders [68]. Metabolic syndrome can be responsible for an elevation in these biomarkers [66], as change in lipid metabolism has important effects on serum ALT levels [69].

### Albumin and stiffness index

A negative relationship was found between serum Albumin and stiffness index, as observed in previous studies [70]. Hypoalbuminemia was significantly associated with inflammatory markers [71], with oxidative stress and with endothelial dysfunction [72] leading to atherosclerosis.

### Cystatin and stiffness index

Cystatin c is a cysteine protease inhibitor and a sensitive marker of the renal function [73]. Cystatin c is a behavioral integrator factor. This cysteine is highly correlated with numerous medical conditions, such as metabolic syndrome, diabetes, physical activity, tobacco smoke, dietary intake and alcohol consumption [74]. Many investigations have observed that Cystatin c level was correlated with AS in general population [75, 76].

### Testosterone and stiffness index

In this study, in the multivariate analysis, a positive relationship was found between serum Testosterone level and stiffness index. However, in this decision model, Testosterone with low levels was present in the accuracy rules for major part of discrimination of arterial stiffness and considered as the last specific factor only for rules 1, 6 and 7. This can explain the inconsistent relation for multivariate analysis observed in the literature. Low serum Testosterone level was associated with aging-related vascular stiffness [77]. But this relationship remains inconsistent, and the underlying mechanism is unclear. This relationship should be investigated in future clinical trials to better understand its interaction.

### Phosphate and stiffness index

Many investigations have associated serum Phosphate with AS [78–80] in participants with CKD or without [78, 81]. The action of serum Phosphate on AS remains unclear. VSMC can retain their mineralized role face to augmentation levels of Phosphate [82]. Phosphate in combination with calcium rates can lead to the death of VSMC and apoptotic body release (with inflammatory processes activation) and matrix vesicle release enhancing calcification mechanism [83]. The increase in Phosphate rates is associated with the diminution of the synthesis of vitamin D, this lead to stimulate arterial calcification [84].

### Limitations

The principal strength of this investigation is the very large sample size of the population. Moreover, the use of the Pulse Trace device to measure AS on account of greater variability in ASI values relative to other available devices [85]. The UK Biobank study presented a few responses rate, which was of 5.5% and potential volunteer bias could be involved. But, given the large sample size and high internal validity of the UK Biobank protocol, these limitations could unlikely to interfere the observed associations [86, 87]. Moreover, the study cohort consisted of middle-aged English participants, thus, the results could not be generalized to other groups of age and ethnic. The UK Biobank was based on standardized protocols to collect anthropometric data. This standardization ensures the replication of data collection regardless of when, where and by whom volunteers are performed. This can add validity to the findings. Statistical significance is correlated to the sample size. Among large sample, the statistical significance between groups could occur with very few differences which are not clinically meaningful. Nevertheless, numerous studies with different sample sizes have shown the interest of these biological parameters, showing the possible interest of such biological factors in CV assessment reports. Participants with reported CV diseases (i.e. heart attack, angina and stroke) in questionnaires and diagnosed by a doctor were excluded. Chronic heart failure was not included in the questionnaires, and participants with it were not excluded from the analyses. This a main limit of this study. ASI values performed in the UK Biobank methodology was not the gold standard used, like cfPWV and is not an accurate measure of central arterial stiffness. This could bias the results observed. Nevertheless, this measurement has been validated by three independent studies comparing with cfPWV. These investigations concluded that both measure measures were mainly correlated [10, 30, 31]. Stiffness index calculation is dependent on the predicted determination, which could be different in other populations and potentially depends on the ASI measurement method performed. The estimation of the predicted ASI value was based on participants without hypertension, diabetes mellitus, previous cardiovascular events and chronic kidney disease, strengthening the individual relevance of stiffness index assessment. However, this “healthy” population presented some health problems, including dyslipidemia, active smoking and sedentary behavior. These lifestyle factors may affect the determination of predicted ASI value, but they are observed in the general population (for dyslipidemia for example) and can thus reflect a predicted ASI value closer to reality. Moreover, the large number of “healthy” participants may enhance the possible generalization of the predicted ASI value calculation. The cross-sectional aspect of the study did not allow presentation of a model which would grow more accurately over time. An external validation is needed on another large database to confirm the accuracy of the model and its significant higher value than multiple logistic regression.

### Conclusion

The study performed a decision tree model to present the different interaction between clinical and blood cutoff parameters associated with arterial stiffness, identified by a positive stiffness index.

Ten independent factors were highlighted in the logistic multivariate analysis. The ten independent classifiers were ranked from the most sensitives: HDL cholesterol≤1.425 mmol/L, smoking pack years≥9.2pack-years, Phosphate≥1.172 mmol/L, to the most specifics: Cystatin c≤0.901 mg/L, Triglycerides≥1.487 mmol/L, Urate≥291.9 *μ*mol/L, ALT≥22.13 U/L, AST≤32.5 U/L, Albumin≤45.92 g/L, Testosterone≥5.181 nmol/L. This work could suggest adding these biological measures in a CV assessment face to an increased AS suspicion and CV risks. Nevertheless, the results observed in this large sample of participants should be investigated in reduced and specific samples and prospective investigations to compare and validate these cutoff values. This first decision tree model remains an experimental model for stiffness index. The interest of this work was to present different classification tools for risk of high stiffness index, an index of arterial stiffness. These decision models provide accurate and useful classification tools for identifying risk associated with arterial stiffness and could be mainly developed in future programs for CV risk prevention.

## Supporting information

S1 Table Collinearities between biological parameters. (DOCX)
