# Supplementary material for: Arterial stiffness and biological parameters: A decision tree machine learning application in hypertensive participants
Source: PLoS One. 2023 Jul 7;18(7):e0288298. doi: 10.1371/journal.pone.0288298 (PMC10328363; doi:10.1371/journal.pone.0288298)
Supplement: S1 Table — (DOCX) [file pone.0288298.s001.docx]

Supplementary table: Collinearities between biological parameters

|  | **ALT** | **albumin** | **Alkaline phosphatase** | **Apo A** | **Apo B** | **ALT** | **Calcium** | **Creatine** | **CRP** | **Cystatine c** | **GGT** | **Glucose** | **HDL chol** | **IGF1** | **LDL** | **Lp(a)** | **Phosphate** | **Testosterone** | **Total bilirubin** | **Total chol** | **Triglycerides** | **Urate** | **Vit D** |
| --- | --- | --- | --- | --- | --- | --- | --- | --- | --- | --- | --- | --- | --- | --- | --- | --- | --- | --- | --- | --- | --- | --- | --- |
| ALT | 1.0000 | 0.1226 | 0.1080 | -0.1507 | 0.0561 | 0.7616 | 0.0700 | 0.0555 | 0.0278 | 0.0340 | 0.4466 | 0.0920 | -0.1961 | -0.0631 | 0.0014 | -0.0254 | -0.0332 | 0.1536 | 0.0769 | -0.0297 | 0.2195 | 0.2269 | -0.0791 |
| albumin | 0.1226 | 1.0000 | -0.0590 | 0.1206 | 0.0801 | 0.0836 | 0.4891 | -0.0099 | -0.1965 | -0.1560 | 0.0423 | -0.0210 | 0.0973 | 0.1409 | 0.0864 | -0.0329 | 0.0923 | 0.0800 | 0.1578 | 0.1113 | 0.0388 | 0.0382 | 0.0339 |
| Alkaline phosphatase | 0.1080 | -0.0590 | 1.0000 | -0.0147 | 0.0381 | 0.1112 | 0.0472 | -0.0386 | 0.1893 | 0.1376 | 0.2257 | 0.0556 | -0.0200 | -0.0320 | 0.0418 | 0.0062 | 0.0184 | -0.1305 | -0.0721 | 0.0437 | 0.0622 | -0.0259 | -0.0879 |
| Apo A | -0.1507 | 0.1206 | -0.0147 | 1.0000 | -0.0406 | -0.0248 | 0.1760 | -0.1928 | -0.0943 | -0.1728 | 0.0077 | -0.0561 | 0.9196 | -0.0500 | 0.0845 | 0.0111 | 0.1177 | -0.2646 | -0.0214 | 0.3299 | -0.2857 | -0.2320 | 0.0686 |
| Apo B | 0.0561 | 0.0801 | 0.0381 | -0.0406 | 1.0000 | 0.0047 | 0.1091 | -0.0596 | 0.0326 | -0.0369 | 0.0503 | -0.0964 | -0.0249 | -0.0170 | 0.9509 | 0.0587 | -0.0016 | -0.0199 | -0.0282 | 0.8753 | 0.2307 | 0.0674 | -0.0806 |
| AST | 0.7616 | 0.0836 | 0.1112 | -0.0248 | 0.0047 | 1.0000 | 0.0656 | 0.0474 | 0.0225 | 0.0480 | 0.4504 | 0.0398 | -0.0514 | -0.1277 | -0.0269 | -0.0229 | -0.0219 | 0.1382 | 0.1083 | -0.0260 | 0.1129 | 0.1783 | -0.0211 |
| Calcium | 0.0700 | 0.4891 | 0.0472 | 0.1760 | 0.1091 | 0.0656 | 1.0000 | -0.0286 | -0.0558 | -0.0020 | 0.0359 | 0.0541 | 0.1557 | 0.0735 | 0.1234 | -0.0048 | 0.1998 | -0.1243 | 0.0335 | 0.1562 | 0.0464 | 0.0448 | 0.0652 |
| Creatine | 0.0555 | -0.0099 | -0.0386 | -0.1928 | -0.0596 | 0.0474 | -0.0286 | 1.0000 | -0.0159 | 0.6630 | 0.0283 | -0.0067 | -0.2073 | 0.1156 | -0.0856 | -0.0067 | -0.0365 | 0.3685 | 0.1072 | -0.1316 | 0.0819 | 0.3787 | 0.0434 |
| CRP | 0.0278 | -0.1965 | 0.1893 | -0.0943 | 0.0326 | 0.0225 | -0.0558 | -0.0159 | 1.0000 | 0.1375 | 0.1049 | 0.0397 | -0.0982 | -0.1313 | 0.0016 | 0.0065 | 0.0009 | -0.0998 | -0.0901 | -0.0225 | 0.0434 | 0.0901 | -0.0537 |
| Cystatine c | 0.0340 | -0.1560 | 0.1376 | -0.1728 | -0.0369 | 0.0480 | -0.0020 | 0.6630 | 0.1375 | 1.0000 | 0.0564 | 0.0052 | -0.2209 | -0.0232 | -0.0715 | -0.0190 | 0.0478 | 0.0875 | 0.0144 | -0.1115 | 0.1382 | 0.3245 | -0.0258 |
| GGT | 0.4466 | 0.0423 | 0.2257 | 0.0077 | 0.0503 | 0.4504 | 0.0359 | 0.0283 | 0.1049 | 0.0564 | 1.0000 | 0.0767 | -0.0456 | -0.1079 | 0.0176 | -0.0247 | -0.0336 | 0.1212 | 0.0564 | 0.0305 | 0.1886 | 0.1883 | -0.0636 |
| Glucose | 0.0920 | -0.0210 | 0.0556 | -0.0561 | -0.0964 | 0.0398 | 0.0541 | -0.0067 | 0.0397 | 0.0052 | 0.0767 | 1.0000 | -0.0805 | -0.0680 | -0.1362 | -0.0127 | -0.1045 | -0.0208 | -0.0108 | -0.1403 | 0.0895 | -0.0126 | -0.0492 |
| HDL chol | -0.1961 | 0.0973 | -0.0200 | 0.9196 | -0.0249 | -0.0514 | 0.1557 | -0.2073 | -0.0982 | -0.2209 | -0.0456 | -0.0805 | 1.0000 | -0.0187 | 0.1177 | 0.0260 | 0.1139 | -0.2748 | -0.0176 | 0.3554 | -0.4470 | -0.3003 | 0.0868 |
| IGF1 | -0.0631 | 0.1409 | -0.0320 | -0.0500 | -0.0170 | -0.1277 | 0.0735 | 0.1156 | -0.1313 | -0.0232 | -0.1079 | -0.0680 | -0.0187 | 1.0000 | 0.0072 | 0.0168 | 0.0532 | 0.0814 | 0.0396 | 0.0005 | -0.0267 | -0.0347 | 0.0617 |
| LDL | 0.0014 | 0.0864 | 0.0418 | 0.0845 | 0.9509 | -0.0269 | 0.1234 | -0.0856 | 0.0016 | -0.0715 | 0.0176 | -0.1362 | 0.1177 | 0.0072 | 1.0000 | 0.0587 | 0.0190 | -0.0572 | -0.0406 | 0.9506 | 0.1584 | -0.0083 | -0.0643 |
| Lp(a) | -0.0254 | -0.0329 | 0.0062 | 0.0111 | 0.0587 | -0.0229 | -0.0048 | -0.0067 | 0.0065 | -0.0190 | -0.0247 | -0.0127 | 0.0260 | 0.0168 | 0.0587 | 1.0000 | 0.0073 | -0.0234 | -0.0142 | 0.0507 | -0.0586 | -0.0204 | -0.0258 |
| Phosphate | -0.0332 | 0.0923 | 0.0184 | 0.1177 | -0.0016 | -0.0219 | 0.1998 | -0.0365 | 0.0009 | 0.0478 | -0.0336 | -0.1045 | 0.1139 | 0.0532 | 0.0190 | 0.0073 | 1.0000 | -0.2224 | -0.0879 | 0.0508 | -0.0122 | -0.0955 | -0.0184 |
| Testosterone | 0.1536 | 0.0800 | -0.1305 | -0.2646 | -0.0199 | 0.1382 | -0.1243 | 0.3685 | -0.0998 | 0.0875 | 0.1212 | -0.0208 | -0.2748 | 0.0814 | -0.0572 | -0.0234 | -0.2224 | 1.0000 | 0.2496 | -0.1320 | 0.0460 | 0.3333 | 0.0498 |
| Total bilirubin | 0.0769 | 0.1578 | -0.0721 | -0.0214 | -0.0282 | 0.1083 | 0.0335 | 0.1072 | -0.0901 | 0.0144 | 0.0564 | -0.0108 | -0.0176 | 0.0396 | -0.0406 | -0.0142 | -0.0879 | 0.2496 | 1.0000 | -0.0676 | -0.0706 | 0.1176 | 0.0610 |
| Total cholesterol | -0.0297 | 0.1113 | 0.0437 | 0.3299 | 0.8753 | -0.0260 | 0.1562 | -0.1316 | -0.0225 | -0.1115 | 0.0305 | -0.1403 | 0.3554 | 0.0005 | 0.9506 | 0.0507 | 0.0508 | -0.1320 | -0.0676 | 1.0000 | 0.1611 | -0.0620 | -0.0552 |
| Triglycerides | 0.2195 | 0.0388 | 0.0622 | -0.2857 | 0.2307 | 0.1129 | 0.0464 | 0.0819 | 0.0434 | 0.1382 | 0.1886 | 0.0895 | -0.4470 | -0.0267 | 0.1584 | -0.0586 | -0.0122 | 0.0460 | -0.0706 | 0.1611 | 1.0000 | 0.2630 | -0.1546 |
| Urate | 0.2269 | 0.0382 | -0.0259 | -0.2320 | 0.0674 | 0.1783 | 0.0448 | 0.3787 | 0.0901 | 0.3245 | 0.1883 | -0.0126 | -0.3003 | -0.0347 | -0.0083 | -0.0204 | -0.0955 | 0.3333 | 0.1176 | -0.0620 | 0.2630 | 1.0000 | -0.0265 |
| Vit D | -0.0791 | 0.0339 | -0.0879 | 0.0686 | -0.0806 | -0.0211 | 0.0652 | 0.0434 | -0.0537 | -0.0258 | -0.0636 | -0.0492 | 0.0868 | 0.0617 | -0.0643 | -0.0258 | -0.0184 | 0.0498 | 0.0610 | -0.0552 | -0.1546 | -0.0265 | 1.0000 |
